# The Bacterial Communities Associated with Honey Bee (*Apis mellifera*) Foragers

**DOI:** 10.1371/journal.pone.0095056

**Published:** 2014-04-16

**Authors:** Vanessa Corby-Harris, Patrick Maes, Kirk E. Anderson

**Affiliations:** 1 United States Department of Agriculture, Carl Hayden Bee Research Center, Tucson, Arizona, United States of America; 2 Center for Insect Science, University of Arizona, Tucson, Arizona, United States of America; Emory University, United States of America

## Abstract

The honey bee is a key pollinator species in decline worldwide. As part of a commercial operation, bee colonies are exposed to a variety of agricultural ecosystems throughout the year and a multitude of environmental variables that may affect the microbial balance of individuals and the hive. While many recent studies support the idea of a core microbiota in guts of younger in-hive bees, it is unknown whether this core is present in forager bees or the pollen they carry back to the hive. Additionally, several studies hypothesize that the foregut (crop), a key interface between the pollination environment and hive food stores, contains a set of 13 lactic acid bacteria (LAB) that inoculate collected pollen and act in synergy to preserve pollen stores. Here, we used a combination of 454 based 16S rRNA gene sequencing of the microbial communities of forager guts, crops, and corbicular pollen and crop plate counts to show that (1) despite a very different diet, forager guts contain a core microbiota similar to that found in younger bees, (2) corbicular pollen contains a diverse community dominated by hive-specific, environmental or phyllosphere bacteria that are not prevalent in the gut or crop, and (3) the 13 LAB found in culture-based studies are not specific to the crop but are a small subset of midgut or hindgut specific bacteria identified in many recent 454 amplicon-based studies. The crop is dominated by *Lactobacillus kunkeei*, and Alpha 2.2 (Acetobacteraceae), highly osmotolerant and acid resistant bacteria found in stored pollen and honey. Crop taxa at low abundance include core hindgut bacteria in transit to their primary niche, and potential pathogens or food spoilage organisms seemingly vectored from the pollination environment. We conclude that the crop microbial environment is influenced by worker task, and may function in both decontamination and inoculation.

## Introduction

The honey bee, *Apis mellifera*, is critical for the pollination of many economically important crops. Continued colony losses have called for a deeper understanding of both symbiotic and pathogenic microbial interactions, particularly as they relate to food storage and the pollination environment. As part of a typical migratory beekeeping operation, the same population of honey bee colonies can be exposed to 5–10 agricultural ecosystems over the course of a year. At each site, foragers encounter a unique set of variables that includes climate, floral components, biocides, and water sources. Many biocides are later found in the wax and stored pollen, or “beebread”, of commercial operations [1], [2]. Evidence suggests that sub-lethal biocide exposure may alter the microbial balance of the individual, hive, or food stores resulting in long-term negative effects [3]–[5]. Recent results from comprehensive molecular surveys suggest the potential for microbial imbalance at many levels of organization [1], [5]–[12], inviting a closer look at a variety of factors that influence the microbial constitution of the colony, particularly the origin and integrity of microbial communities in the gut, crop, and food stores.

The honey bee colony “superorganism” consists of individual, group, and hive components, complete with a large repertoire of socially interactive and homeostatic behaviors [13]. Anatomically, the foregut (hereafter crop) is the honey bee's social/nutritional interface. This portion of the alimentary tract is essentially an inflatable storage bag used to transport nectar from the flower to the hive, share liquid nutrition with sibling nestmates, and selectively pass pollen into the midgut [14]. More generally, the crop represents the microbial intersection of food sharing, food storage and the pollination environment. At the flower, pollen foragers use their forelegs and tongue to gain access to nectar and pollen. Pollen accumulated on the head and body hairs is consolidated using the forelegs, mixed with liquid sugars from the crop, and this sticky mixture is packed into hindleg pollen baskets called corbiculae. Once returned to the hive, corbicular pollen pellets are packed tightly into wax cells and become beebread, a nutrient dense mixture of pollen, honey and various microbes. Beebread has been likened to a silage environment wherein the protoplasmic nutrients locked within the durable pollen coat are fermented, or essentially “pickled” for future consumption [15]–[17]. Pollen storage is critical because stored food provides the honey bee with essential nutrients during the winter or periods of pollen dearth.

The present understanding of crop bacteria is culture-dependent, and maintains that “13 different beneficial bacteria reside inside the honey crop of bees, are placed on pollen at the flower, and work synergistically to protect beebread from degradation” [18]–[24]. These conclusions are provisional however, as they include culture bias and highly inconsistent detection of the 13 proposed strains, all labeled “lactic acid bacteria” (LAB) although four of the strains belong to the genus *Bifidobacterium*. An alternative hypothesis states that these 13 “crop-specific” LAB are not specific to the crop but are instead part of a larger subset of bacteria occurring preferentially in other gut compartments or hive microenvironments [17]. Newly emerged bees are seemingly free of bacteria, and appear to acquire their core hindgut bacteria from the hive environment, food stores, or older individuals [17], [25]. Combined with the fact that honey bees are constantly swapping food via oral trophallaxis [26], bacteria evolved to occupy the midgut, hindgut or food storage niche would likely occur with some frequency in the crop of most bees.

Recent non-culture based investigations of the honey bee microbiome have focused on bees captured from within the hive, sampling either the entire alimentary tract, or only the midgut and hindgut [25], [27]–[35]. Less attention has been paid to the microbial diversity of foraging bees, food stores and the pollination environment. Independent findings from solitary and social pollinators suggest that both potentially pathogenic and beneficial microbes are regularly vectored from the pollination environment or floral sources [7], [36]–[39]. Some sources of floral nectar contain bacteria similar to those found in the stored food or hive materials of honey bees, suggesting that the crop acts as a “semi-permeable filter”, a selective environment wherein particular bacteria can survive to be vectored between floral and hive environments [17], [40]–[50].

Here we investigate the crop and gut microbial diversity of returning pollen foragers and the corbicular pollen they collected using 454 amplicon assays of the 16S rRNA gene. To determine the origin of the bacterial communities on inbound pollen, we compare forager gut and corbicular pollen communities for two seasonal time points in the same geographic location. We also test the hypothesis that bacteria associated with the honey bee crop and placed on corbicular pollen are composed of the 13 putative core crop bacteria. We compare our results for known pollen foragers with past studies of gut microbial communities of typically younger in-hive bees.

## Methods

### Ethics Statement

All colonies were sampled from apiaries located at the USDA Carl Hayden Bee Research Center in Tucson AZ. Our field collections did not involve endangered or protected species and no specific permissions were required because the study was conducted by USDA employees.

### Bee samples and DNA preparation

Pollen foragers were collected in the fall (3 December 2012) and spring (20 March 2013) from two colonies housed side by side in the same apiary at the USDA Carl Hayden Bee Research Center in Tucson, Arizona. In December, the availability of pollen sources had decreased and the colonies had ceased brood rearing and were storing pollen for the winter. In contrast, colonies were actively brood rearing in March, collecting pollen from a variety of different plant sources and quickly converting this pollen into new brood. For each colony collection, the hive entrance was blocked and returning pollen foragers were collected in less than 5 minutes per hive. In both the spring and fall, fourteen individual pollen foragers per colony were captured with sterile soft forceps, placed in sterile Falcon tubes, and chilled on ice. Pollen loads were removed from both corbiculae (i.e., pollen baskets) before dissecting each bee's GI tract, containing the entire length of the alimentary tract from the crop through to the rectum. To compare crop and corbicular pollen diversity, additional pollen foragers were sampled similarly in the spring from 14 different colonies in the apiary. Initial samples composed of individual crops produced negligible template DNA and inconsistent PCR products. Thus, the crops and corbicular pollen of ten pollen foragers per colony were pooled, yielding 14 libraries composed of 10 crops each, and 14 libraries composed of the associated corbicular pollen from those same 10 foragers (10 foragers×2 legs = 20 corbicular loads per library).

Total genomic DNA was extracted from individual GI tract samples, pooled crop samples, and pooled and individual corbicular pollen samples. Prior to DNA extraction, gut and crop samples were processed similarly by bead beating the tissue for 30 sec in a sterile 2 mL centrifuge tube containing 350 µl of 0.5 mm silica beads and 1.5 ml TE/Triton × lysis buffer (20 mM Tris-HCl, 2 mM EDTA, 1.2% Triton X-100, pH 8.0). The supernatant was transferred to a new 1.5 ml centrifuge tube and centrifuged for 30 minutes. The supernatant was removed and 180 µl TE/Triton × lysis buffer (20 mM Tris-HCl, 2 mM EDTA, 1.2% Triton X-100, pH 8.0; 20 mg/ml lysozyme added immediately before use) was added. Upon removal from the bee, corbicular pollen was added to 1.5 ml of TE/Triton × buffer (20 mM Tris-HCl, 2 mM EDTA, 1.2% Triton X-100, pH 8.0) and vortexed for 5 minutes. Each sample was briefly centrifuged at low speed and the supernatant was transferred to a new 2 ml bead beating centrifuge tube where it was centrifuged for 10 minutes on high to pellet the bacterial cells. This wash cycle was repeated 4 times to maximize the amount of bacterial detachment from the pollen grains. After the final wash cycle 350 µl of 0.5 mm silica beads were added to the 2 ml tube and bead beaten for 30 sec. The supernatant was then transferred to a new 1.5 ml centrifuge tube and centrifuged for 30 minutes. The supernatant was removed and 180 µl TE/Triton × lysis buffer (20 mM Tris-HCl, 2 mM EDTA, 1.2% Triton X-100, pH 8.0; 20 mg/ml lysozyme added immediately before use) was added. Samples in lysis buffer were then subjected to genomic DNA extraction using the GeneJet Genomic DNA Purification Kit (Fermentas) following the protocol for gram-positive bacteria.

### PCR and pyrosequencing

The V1-V2 region of the 16S rRNA gene of the samples was PCR amplified using universal 16S rRNA primers fitted with 454 FLX Titanium adapter sequences (27F 5′-CCATCTCATCCCTGCGTGTCTCCGACTCAG-NNNNNNNNNN-agagtttgatcctggctcag -3′; 338R: 5′- CCTATCCCCTGTGTGCCTTGGCAGTCTCAG-tgctgcctcccgtaggagt -3′; uppercase letters denote the adapter sequences, N's indicate library-specific barcodes, lowercase letters indicate universal 16S rRNA primers). Amplicons were sequenced using Roche 454 GS FLX Titanium sequencing.

### Pyrotagged sequence analysis

Sequences were processed using mothur v.1.26.0 [51]. Sequences in the.sff files were quality filtered using the trim.flows command (minflows = 360, maxflows = 720) and all sequences less than 150 base pairs (bp) with more than 2 base mismatches to the 27F primer sequence or 1 mismatch to the 10 bp pyrotag after trimming were eliminated using the trim.seqs command. Pyrotags were removed and the sequences were aligned to Silva SSURef database (v102) using the align.seqs command. Sequences that started after the 27F sequence or that were shorter than the 98% of the sequences were eliminated using the screen.seqs command. Sequences that were within 1% similarity were clustered together using the pre.culster command. Chimeras were removed using UCHIME [52] and any sequences that were of mitochondrial, chloroplast, Archaeal, Eukaryote, or unknown origin were removed. The sequence libraries were then concatenated and aligned as described above. A distance matrix was constructed for the aligned sequences using the dist.seqs command and the default parameters. Sequences were then binned into operational taxonomic units (OTUs) based on 97% sequence similarity. It should be noted that our investigation did not warrant a single experiment-wide cutoff for delineating taxonomy. Honey bee gut taxonomy has been refined by many recent papers [28]–[35], but there is far less taxonomic information concerning bacterial communities associated with the hive or general pollination environment. Representative sequences from each 97% OTU were characterized in two ways. First, these sequences were classified with the RDP Naïve Bayesian Classifier using a manually constructed training set that contained sequences from the greengenes 16S rRNA database (version gg_13_5_99 accessed May 2013), the RDP version 9 training set, and all full length honey bee associated gut microbiota listed in NCBI (accessed July 2013) trimmed to the V1-V2 region of the 16S rRNA gene. Next, representative OTU's classified by RDP were then subject to a BLAST query using the NCBI nt database, taking the hit with the lowest e-value less than or equal to 1×10^−10^. Any remaining sequences that were of chloroplast or mitochondrial origin were removed as well as sequences classified with less than 100% confidence at the Phylum level using the RDP Naïve Bayesian Classifier [53]. Rarefaction curves were generated for each of the libraries using the rarefaction.single command. For sample types that were completely or near completely sampled (according to the rarefaction curves generated), individual libraries were pooled by sample type (i.e., fall guts or spring guts) and Chao estimates of species richness and Good's estimate of coverage were determined using the summary.shared command.

### Data accessibility

Sequences can be found in the NCBI Sequence Read Archive (SRA) under accession number SRP035369. Table S1 links the pyrosequencing barcodes to each library consisting of gut, crop or corbicular pollen samples from individual bees or bees pooled by colony.

### Analyses of crop- and gut-associated “lactic acid bacteria” (LAB)

The present paradigm claims that the crop contains 13 strains of “Lactic Acid Bacteria” (LAB; 9 *Lactobacillus* spp., 4 *Bifidobacterium* spp.) that are core to the crop, that the bee deposits on corbicular pollen at the flower, and that act in synergy to preserve beebread [18]–[24]. An alternate hypothesis is that most of LAB found in the crop are simply in transit to the hindgut [17]. We tested these conflicting hypotheses in two ways. First, we conducted a BLAST search using full-length sequences of the 16S rRNA gene from these 13 LAB as query sequences and the sequences found in the fall gut, spring gut, and spring crop libraries. The BLASTn algorithm was used (-task = blastn) and successful hits were those with an e-value≤1×10^−10^ and 100% sequence identity for the length of the alignment. Tallies were taken to determine the number of 100% hits to the 13 LAB to determine whether these bacterial strains could be present in our samples and whether they were more prevalent in the crop than the guts. The majority of sequences that were significant hits to these 13 LAB matched the *Lactobacillus* spp. but the number of *Bifidobacterium* spp. hits was negligible (see Results). We next constructed a phylogeny to examine the similarity of the *Lactobacillus* sp. isolated from the pooled crops and individual guts of spring or fall bees amongst themselves and to known sequences of *Lactobacillus* spp. A sequence database was constructed using 16 *Lactobacillus* OTUs from the present study, 9 putative crop-specific *Lactobacillus* sequences, and 27 published full-length *Lactobacillus* sp. 16S rRNA sequences as a reference. These published full-length sequences, and the sequences identified as *Lactobacillus* spp. in the present study were aligned using Muscle v3.8.31 [54] and manually edited for quality using BioEdit [55]. The alignment was then cropped to include only the V1/V2 region, and gaps were eliminated, leaving 272 positions in the final alignment. A Neighbor-Joining phylogeny was built using MEGA version 5 [56] and 500 bootstrap replicates. The analysis included 52 nucleotide sequences and all codon positions were included. Rate variation among sites was modeled with a gamma distribution. We used this phylogeny to illustrate whether the crop-associated bacteria were distinct from the gut-associated bacteria found in this and other studies.

### Crop plate counts

We determined by direct plate count the number of bacteria in the crops of bees performing different tasks because (1) there are stark differences concerning the predicted number of bacteria in the crop as defined in the literature [18], [22], [25], and (2) we experienced consistent difficulties extracting sufficient quantities of bacterial DNA and/or performing PCR amplification of crop bacteria from individual crops. Combined with the reliable amplification of the positive PCR control (previously verified *Apis mellifera* gut sample), this indicated relatively low numbers of bacterial cells in crop samples. In-hive nectar processors, nurse bees, general foragers, and pollen foragers were collected in the spring of 2012 from a three frame observation hive separate from the colonies used to gather the pyrosequencing data. In-hive nectar processors were defined as bees that made 5 consecutive visits to stored honey in the hive. Nurses were bees that made 5 consecutive visits to developing larvae. General foragers were bees returning from flights that did not have pollen in their pollen baskets, and pollen foragers had pollen loads in their corbiculae. Individuals were collected into sterile Falcon tubes on wet ice and brought to the lab for immediate dissection. The crops of 12 nectar processors and 12 nurse bees were dissected and their contents were recorded (full, empty, or half full). Most of the nectar processors' crops were empty and most of the nurse crops were full so for consistency only these samples were used in the analysis (nectar processors N = 9, nurses N = 10). The crops of 12 general foragers and 12 pollen foragers were dissected and weighed. Each crop was homogenized in 1 ml of physiological saline and 100 µl was plated onto deMan-Rosaga-Sharp (MRS) and Tryptic-Soy agar (TSA) plates and placed in aerobic or microaerophilic (5% CO_2_) conditions at 35°C for 24 h. Counts of the number of colony forming units (CFUs) per plate were made and, where appropriate, the log number of CFUs per gram of crop was calculated to compare to other studies [47], [57]. Comparisons were made between CFUs growing in aerobic versus microaerophilic conditions for each media type or between the CFUs growing on MRS versus TSA plates incubated in similar environmental conditions.

## Results

### Reads analyzed and taxa generated

A total of 1,616,883 reads were generated across the six sample types and 1,452,224 reads remained after the initial quality trimming (Table 1). On average, 1.4% of these reads were chimeric and were removed. There were large differences among sample types in the number of reads that were chloroplast in origin (cpDNA). Of the gut and crop samples, an average of 2.6% of the remaining non-chimeric reads were cpDNA, while 87% of the corbicular pollen reads were cpDNA. This cpDNA contamination resulted in drastically different average read numbers for the crop and gut libraries compared to the corbicular pollen libraries. A total of 812 operational taxonomic units (OTUs) were resolved across the six sample types at the 97% level of similarity (Table S2). BLAST results from the NCBI nucleotide database were in complete agreement with the RDP classifier result, which suggested that the RDP classification was adequate for grouping the taxa as gut microbiota or otherwise as presented in the tables and figures. Rarefaction curves generated for each sample type and library show that the diversity of the crop and gut samples was completely or near completely sampled, the diversity of the spring corbicular pollen samples was not completely sampled, and the diversity of the fall corbicular pollen samples was near completely sampled (Table S2, Fig. S1). Chao estimates of species richness and coverage were calculated for the four sample types that were completely or near completely sampled (i.e., the crop, fall guts, spring guts, and fall corbicular pollen). Coverage was above 99% for all four sample types and the richness of the fall corbicular pollen was significantly greater than the fall guts, spring guts, and spring crops, which all had comparable species richness (Table 2).

**Table 1:** Summary of pyrotagged sequence processing.

season	tissue or substrate	Total libraries	N^A^ initial reads	N after quality trimming	% chimeras	N after cpDNA^B^ removed	% cpDNA	N after manual culling^E^
fall	corbicular pollen	14^C^	285,365	257,832	0.23	58,218	77	33,284
fall	guts	14^C^	251,856	229,503	0.24	228,563	0.17	191,037
spring	corbicular pollen	14^C^	288,351	259,928	0.12	30,680	88	5,428
spring	guts	14^C^	298,257	267,324	6.4	249,893	0.11	163,468
spring	corbicular pollen	14^D^	245,430	216,426	0.10	9,175	96	4,879
spring	crop	14^D^	247,624	221,211	1.5	201,122	7.5	195,264

A Number of reads produced by the 454-instrument.

B Chloroplast DNA (cpDNA).

C Each library contained material from one individual forager.

D Contained 10 pooled individuals per library. Each library represents a single colony.

E Sequences that were not cpDNA or mitochondrial DNA in origin, and classified at a confidence level of 100% at the Phylum level according to the RDP classifier.

**Table 2:** Estimates of species richness and coverage for adequately sampled libraries.

Sample type	OTUs	Good's Coverage	Chao estimate of species richness	LCI^A^	HCI^A^
Fall corbicular pollen	630	0.994	813.82	753.99	902.52
Fall guts	82	0.999	130.33	100.91	205.49
Spring guts	85	0.999	99.44	90.15	125.46
Spring crops	116	0.999	159.05	135.87	209.26

A Low (LCI) and high (HCI) 95% confidence intervals for the Chao estimate of species richness. Libraries were randomly sampled in equal numbers for 1000 replicates.

**Figure 1:**
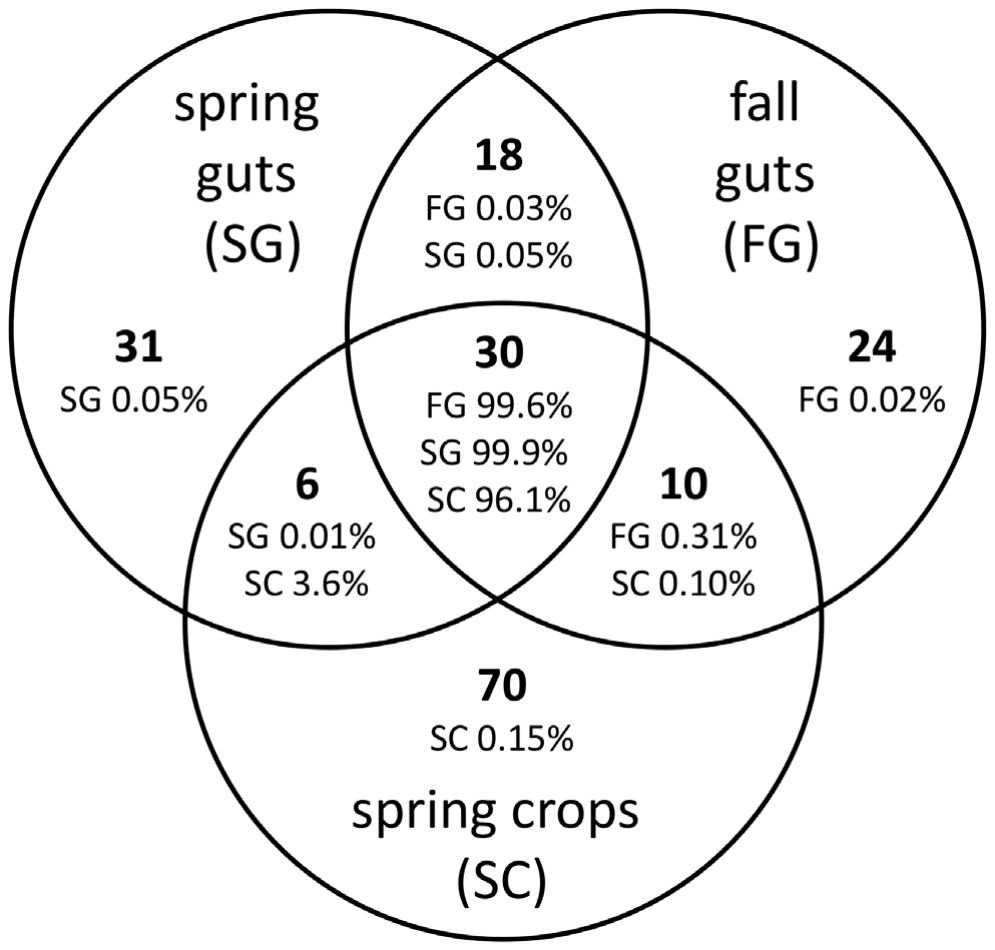
Bacterial taxa shared among the pyrosequencing libraries from the gut and crop samples. Libraries were pooled by sample type (fall gut, spring gut, spring crop) and the number of OTU's (in bold) was defined based on ≥97% sequence similarity. The number of sequences in each of the gut and crop sample sets was calculated for each OTU and the percentage of sequences in each sample type that was either shared among sample sets or unique to a particular sample set was calculated. For example, the 70 OTU's unique to the spring crop samples account for only 0.15% of the total spring crop sequences, while the 30 OTU's shared among all sample sets includes 96.1% of all spring crop sequences.

Venn diagrams comparing the fall gut, spring gut, and spring crop libraries showed that 30 OTUs were shared among all sample types and 70 were specific to the crop (Fig. 1). Moreover, the shared sequences comprised 99.6%, 99.9%, and 96.1% of the fall gut, spring gut, and spring crop sample types, respectively (Fig. 1). The majority of these shared sequences are considered core gut bacteria. Crop-specific taxa were comprised mostly of singletons or OTUs with few sequences (4.16±9.42 s.d. sequences per crop-specific OTU; Table S2). The most abundant OTU found only in the crop contained just 72 sequences and was closely related to *Peptoniphilus* sp., a gram-positive anaerobic coccus that has been found in both floral nectar and on the surface of honey bees [58].

**Figure 2:**
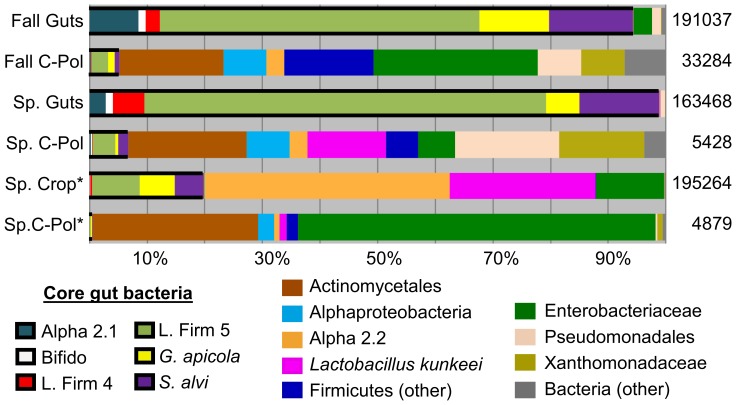
Relative abundances of bacterial groups from each sample type. Libraries (individuals or colonies) were pooled by sample type and the number of sequences belonging to each taxon relative to the total number of sequences in that sample type was determined. Black boxes around portions of each bar denote core gut bacteria. Asterisks (*) denote pooled colony samples, and total reads post filtering are displayed to the right of each sample type.

**Figure 3:**
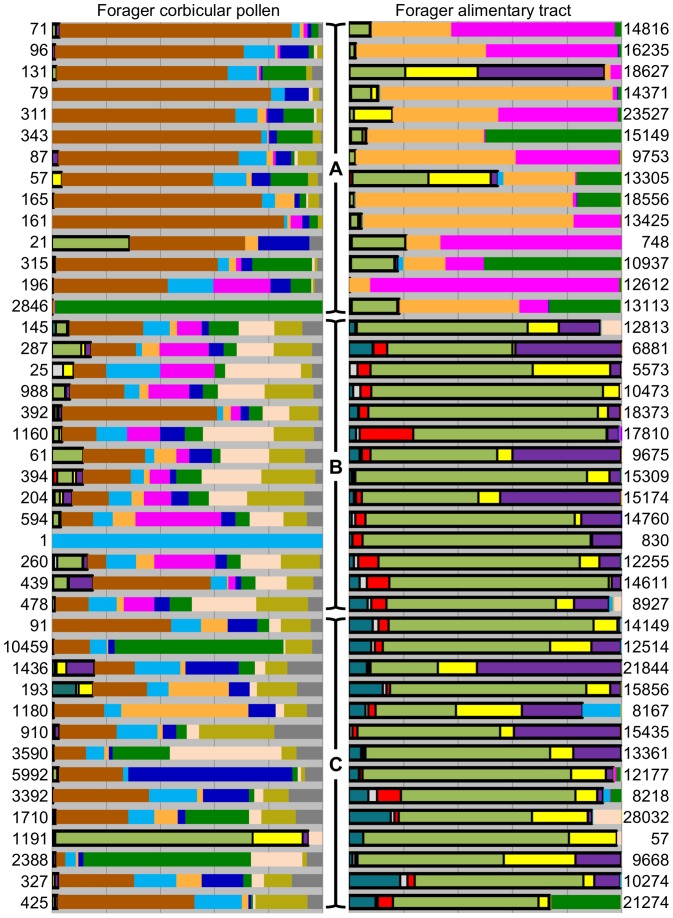
Relative abundances of bacterial groups within each individual library by sample type. Color key is the same as figure 2. In the left column, corbicular pollen libraries were derived from the same bee(s) depicted in the adjacent alimentary tract column. Bars indicated by “A” each denote 10 pooled spring crops and associated corbicular pollen from 14 pooled colony samples; bars within “B” are 14 individual forager guts and their associated corbicular pollen sampled from a single colony in spring, and “C” is 14 individual forager guts and their associated pollen sampled from a single colony in fall. Black boxes around portions of each bar denote the core gut bacteria, and each bar represents a library flanked by its total read number.

The majority of the fall gut (94%) and spring gut (99%) sequences were comprised of core gut bacteria (Figs. 2 and 3). The entire core gut microbiota (Acetobacteraceae (Alpha 2.1), *Lactobacillus* sp. (Firm 4), *Lactobacillus* sp. (Firm 5), *Frischella perrara* (Gamma 2), *Gilliamella apicola* (Gamma 1), *Snodgrassella alvi* (Beta), and a honey bee associated *Bifidobacterium* sp. [30], [59], [60]) was represented to some degree in at least one of the sample groups. Compared to previous studies of in-hive bee guts employing a similar approach [25], [32], [33], we observed more Alpha 2.1 (Table 2; fall guts: 8.5%, spring guts: 2.0%), and more *S. alvi* sequences (Table 3; fall guts: 14.5%, spring guts: 13.8%). Only two *F. perrara* sequences were observed across all sample types (Table 3; fall guts: 0.0005%, spring guts: 0.0006%). Twenty percent of the sequences found in the crop were previously defined as core gut microbiota (Figs. 2 and 3). There was some variation among individuals' guts in the degree that the core was represented (Fig. 3). Most guts were comprised almost entirely of core gut taxa, but this core was less abundant in a few individuals. Where the core taxa did not dominate the whole gut, the remainder of the library was comprised of sequences belonging to the Enterobacteriaceae, Pseudomonadales, or Alpha 1, a relative of *Bartonella* spp. identified previously from bees (Fig. 3).

**Table 3:** Relative abundance^A^ of the core microbiota^B^ from honey bee guts and crops.

Study	Site^C^	Sample	Life stage	Total n sequences	A2	BT	G1	G2	F4	F5	BF	Other^D^
Martinson *et al*. 2011	AZ	whole bee	in hive	271	1.1	11.1	11.8	0.0	10	63.8	0.7	1.5
Martinson *et al*. 2011	AZ	pooled guts	in hive	267	0.0	3.7	9.7	0.0	10.5	60.7	13.1	2.2
Martinson *et al*. 2012	AZ	dissected guts	9d old nurses (N = 3)	78,595	0.0	20.0	12.0	17.0	0.2	49.0	0.8	0.1
Martinson *et al*. 2012	AZ	dissected guts	30d old forager (N = 1)	17,910	0.0	19.8	0.11	57.0	0.32	22.0	0.6	0.0
Sabree *et al*. 2012	MA	dissected guts	12 days old	106,344	1.2^E^	6.7	49.1	1.1	11.1	21.0	5.4	5.2
Moran *et al*. 2012	AZ & MD	dissected guts	in hive, outer frames	329,550	1.0	9.1	11.9	2.0	23.2^F^	45.4^F^	5.4	1.9
present	AZ	dissected guts	pollen forager (fall)	191,037	8.5	14.5	12.1	0.0^G^	2.5	55.4	1.2	5.8
present	AZ	dissected guts	pollen forager (spring)	163,468	2.8	13.8	5.8	0.0^G^	5.5	69.6	1.2	1.3
present	AZ	dissected crops	pollen forager (spring)	195,264	0.0	5.1	6.4	0.0	0.3	8.6	0.1	79.5

A Values rounded to the nearest tenth of a percent.

B A2 is Alpha 2.1; Acetobacteraceae, BT is *Snodgrassella alvi* [60], G1 is *Gilliamella apicola* [60], G2 is *Frischella*.

*perrara* [59], F4 and F5 are *Lactobacillus* species Firm4 and Firm5, and BF is *Bifidobacterium*.

C Site corresponds to U.S. state; AZ = Arizona, MA = Massachusetts, MD = Maryland.

D Bacteria not found consistently across published studies when sampling the entire alimentary tract or the mid/hindgut (see text).

E 1.2% of the library was found to contain Alpha 2.1 when smaller amplicon lengths were allowed.

F Values for *Lactobacillus* sp. Firm 4 and Firm 5 [33] were switched in [32] and are corrected in this table.

G One sequence was found in each sample type.

The crop harbored a microbiota highly distinct from the guts (Figs. 2 and 3). Crop bacteria were dominated by Alpha 2.2 (42%) and *L. kunkeei* (25%; Fig. 2). There was considerable variation in the distribution and composition of the microbial communities among libraries, and crops often contained high levels of Enterobacteriaceae (Fig. 2). The most numerous core gut sequences found in the crop were *Lactobacillus* sp. Firm 5 (8% of sequences), *G. apicola* (6% of sequences), and *S. alvi* (5% of sequences; Fig. 2 and Table S2).

Corbicular pollen contained a diverse microbiota dominated by non-gut and non-crop bacteria including Actinomycetales, Alphaproteobacteria, Enterobacteriaceae, Pseudomonadales, Firmicutes, and Xanthomonadaceae, (Figs. 2 and 3). Much like the crop libraries, there was considerable variation in taxon richness and abundance among corbicular pollen carried by individual foragers (Fig. 3). Libraries were not consistently dominated by one taxon, but Actinomycetales was abundant in the majority of corbicular pollen samples (Fig. 3 and Table S2).

### Similarity of reads to the 13 putative crop-specific “lactic acid bacteria” (LAB)

We examined two alternate hypotheses: (1) the crop harbors 13 specific strains of LAB (9 *Lactobacillus* and 4 *Bifidobacterium* species) that act in synergy to preserve hive food stores [18]–[24], and (2) bacteria specific to the hindgut are frequently sampled from the crop due to high rates of trophallaxis among individuals and the incidental movement of bacteria from the hive environment through the crop on their way to the hindgut [17], [61]. To determine the number of sequences from the crop and gut that are a significant match to the putative 13 crop-specific strains, we performed BLAST searches between the fall gut, spring gut, and spring crop samples and a reference database containing these 13 bacterial strains (Table 4). Two of the *Lactobacillus* strains were not detected in any sample type. Sequences corresponding to the four *Bifidobacterium* strains were uniformly rare, on average representing<0.01% of all alimentary tract sequences combined. Among the nine queried *Lactobacillus* spp., only the sequence corresponding to strain Hma11 (within OTU050) occurred with noticeable frequency in both the gut and crop samples (Table 4). Sequences matching *L. kunkeei* strain Fhon2 were abundant in the crop, but virtually absent from the gut samples (Table 4). Of all sequences classified within the broader taxonomy of *Lactobacillus* Firm 4 or Firm 5, only 1.7% were detected in the crop and matched the putative crop-specific strains. From this same group however, 12.5% of sequences matched the putative crop-specific strains and were found in the gut. Similarly, of all sequences classified as *Bifidobacterium*, only 1.3% matched the putative crop-specific strains and were found in the crop, while 20.8% of all *Bifidobacterium* sequences matched the putative crop-specific strains and were found in the gut.

**Table 4:** Number of sequences from the guts and crops of pollen foragers showing 100% sequence similarity to the 13 putative crop-specific bacterial strains.

Strain A	Group B	Accession number	Sample type^C^					
			Fall guts		Spring guts		Spring crops	
***Lactobacillus*** ** spp.**								
Hma8	A (Firm 5)	EF187243.1	227	0.1	7	0.004	15	0.008
Bma5	A (Firm 5)	EF187242.1	2,297	1.2	1,877	1.2	489	0.2
Biut2	A (Firm 5)	EF187241.1	3,688	1.9	4,282	2.6	116	0.06
Hma2	A (Firm 5)	EF187240.1	0	0.0	0	0.0	0	0.0
Hma11	A (Firm 5)	EU753689.1	11,535	6.0	3,259	2.0	3,471	1.8
Bin4	B (Firm4)	EF187245.1	703	0.4	2,301	1.4	201	0.1
Fhon2	C (*L. kunkeei*)	EF187239.1	88	0.05	118	0.07	37,001	18.9
Fhon13	C (*L. kunkeei*)	HM534758.1	0	0.0	0	0.0	0	0.0
Hon2	D (Firm 4)	EF187244.1	0	0.0	1,319^D^	0.8	0	0.0
***Bifidobacterium*** ** spp.**								
Hma3	n/a	EF187236.1	24	0.01	55	0.03	0	0.0
Bma6	n/a	EF187237.1	118	0.06	274	0.2	36	0.02
Bin7	n/a	EF187234.1	121	0.06	185	0.1	14	0.007
Bin2	n/a	EF187231.1	40	0.02	104	0.06	8	0.004

A Strain names according to [18]–[23].

**Figure 4:**
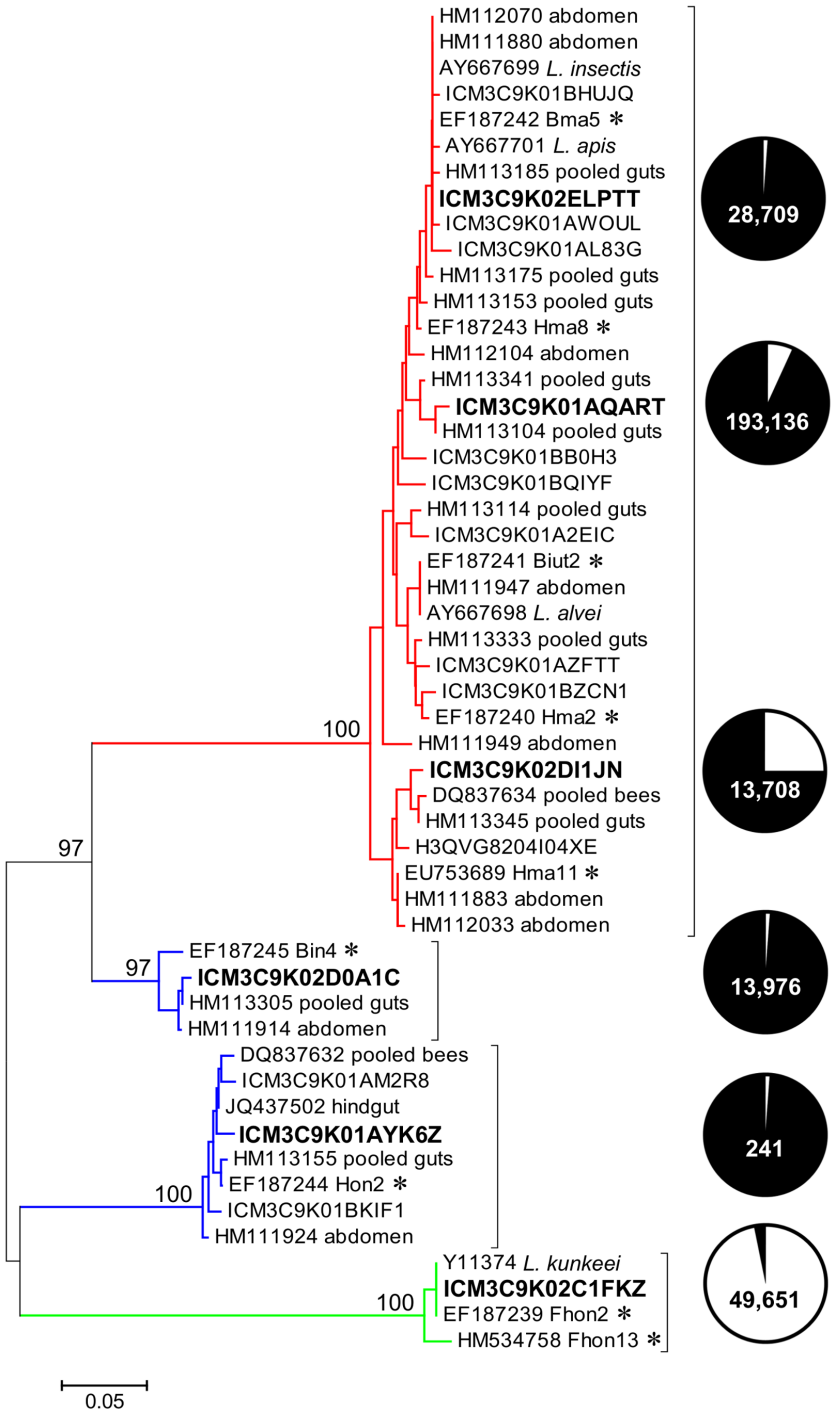
Phylogenetic tree of *Lactobacillus* spp. identified from the guts and crops of foragers. A Neighbor-Joining phylogeny was constructed using the V1/V2 region of the 16S rRNA gene of *Lactobacillus* spp. from alimentary tracts in this study. Published full-length *Lactobacillus* spp. 16S rRNA sequences from gut samples, and putative crop specific sequences (marked with an asterisk) were used for reference. Numbers at the branch nodes indicate the level of bootstrap support for 500 replicates. Representative sequence titles for each OTU (97% sequence similarity) identified in the present study are shown in bold font. Values within pie charts are the number of sequences comprising each of the six *Lactobacillus* OTUs containing more than 100 reads. Pie charts represent the proportion of reads in each OTU occurring in the gut (black) vs. the crop (white). Colored clades correspond to the four major groups identified in Table 4. Please note that the two Firm 4 clades typically form a single clade in phylogenies constructed from full-length 16S rRNA sequences.

B Refers to the 4 clades depicted in Figure 4.

C The first column under each sample type represents the total number of reads from all 14 libraries having 100% sequence similarity to the queried strain. The second column shows the percent of the total sequence reads from that sample type matching the queried LAB strain sequence at 100%.

D Value attained when comparing all trimmed sequences passing quality filtering, but classified at a confidence level <100% at the Phylum level according to the RDP classifier.

A Neighbor-Joining phylogeny of the *Lactobacillus* species identified in our samples revealed 4 distinct groups (Fig. 4). The largest group was closely related to *Lactobacillus* sp. Firm 5. *Lactobacillus* sp. Firm 4 resolved into 2 well-supported clades, while *L. kunkeei* formed its own group (Fig. 4). Again only one sequence matching the putative core crop bacteria [18]–[24], *L. kunkeei* strain Fhon2, was prevalent in the crop (Table 4). After super-imposing these groups onto the sequence tallies, the spring crops were comprised primarily of sequences related to *L. kunkeei*, while the spring and fall guts were populated mostly by *Lactobacillus* Firm4 and Firm5 (Fig. 4).

### Crop counts

Crops of honey bees performing different behaviors were plated as a dilution series on acidic (MRS) and neutral (TSA) media in aerobic or microaerophilic conditions. Nearly all colonies that grew on the plates were indistinguishable according to size, color and colony morphologies. Overall, foragers contained a crop microbiota that flourished in both ambient and microaerophilic atmosphere on both MRS and TSA media (Table 5). In general, bees performing tasks in the hive had substantially less crop bacteria than presumably older foragers. Under ambient atmosphere, the acidic MRS media harbored significantly more CFUs than the neutral TSA media for all honey bee types. Under microaerophilic conditions, more bacterial growth was supported on the acidic media compared to the neutral media, but the difference was non-significant. After adjusting the CFUs for the amount of crop plated (1/10 of each crop plated, see methods; Table 5), the number of CFUs in the crop was many orders of magnitude less than counts reported for the midgut (>10 log CFUs per gram) and rectum (>10 log CFUs per gram) of summer and winter bees [47], [57] and less than the log 6 CFUs in the honey crop reported in [22]. However, the number of CFUs observed here was comparable to the number of CFUs in honey bee crops reported in [18].

**Table 5:** Bacterial counts (CFUs)^A^ for honey bee crops^B^ cultured under different conditions.

Growth conditions		Workers sampled inside the hive		Workers sampled outside the hive	
Media^C^	Atm^D^	Nectar^E^ (9)	Nurses^F^ (10)	General (12)	Pollen (12)
MRS	O_2_ A	0.69±0.85	3.60±1.31	11.93±6.40	7.18±5.54
MRS	CO_2_	1.97±1.06	3.99±1.49	8.75±4.90	6.78±4.39
TSA	O_2_	0.013±0.012	0.026±0.009	3.26±1.45	2.11±1.37
TSA	CO_2_	0.83±0.52	2.21±0.96	3.41±1.21	5.63±3.56

A Mean ± SE colony forming units per crop; multiply listed values by 10^5^,

B Crop is the foregut or social stomach. Sample size in parentheses.

C MRS: de Man, Rogosa and Sharpe media; pH 5.7, TSA: Tryptic soy agar; pH 7.3

D Atmosphere; O_2_  =  Ambient conditions, CO_2_  =  Ambient conditions containing 5% CO_2_.

E Workers with empty crops processing nectar.

F Workers with full crops attending to brood.

## Discussion

Food storage is a critical fitness component of perennial social insects [62]. As a step towards understanding the microbial succession that occurs during pollen storage in the honey bee, we sought to differentiate between bee-mediated microbial inoculations occurring at the flower and microbes introduced as a consequence of environmental exposure. We captured inbound pollen foragers and sequenced the bacterial DNA from individual guts and pooled foreguts (crops), and from the associated corbicular pollen on their hind legs using high throughput sequencing. Our results do not support the presence of 13 core crop “LAB” bacteria and associated beebread preservation proposed by previous culture dependent studies [18]–[24]. Similar to acidic and sugar rich environments of fermented food and silage, the crops of foragers were dominated by *Lactobacillus* and Alpha 2.2 (Acetobacteraceae), but also contained a small number of sporadically abundant Enterobacteriaceae that likely have their origins in the pollination environment. We found that bacteria considered core to the midgut and hindgut can be found in the crops of pollen foragers, but on average, less than 4% of the bacteria identified from corbicular pollen can be considered core gut bacteria. Similarly, 12 of 13 putative crop-specific LAB strains [18]–[24] are simply a subset of the core gut bacteria, occur at low frequency in the crop and gut, and are not placed on corbicular pollen at the flower. The majority of bacteria found in corbicular pollen does not originate with the crop or gut, but appears to originate from the pollination environment.

We used a combination of 16S rRNA gene sequences and culture-based methods to determine the diversity and abundance of bacteria found in the crop, entire alimentary tract and associated corbicular pollen. Sampled microenvironments differed for both the richness and abundance of bacterial taxa (Fig. 2). Data from the entire alimentary tract (guts) was similar across the two sampled time points, and confirms previous definitions of the core gut microbiota based on in-hive bees [25], [29], [30], [32], [33], [35], but reveals differences in relative taxon abundance which may reflect a lack of pollen in the forager diet. Despite far less sequence coverage for corbicular pollen libraries, they were much more diverse than libraries of forager guts and crops (Table 2). Although we used different primers, tissues and methods (i.e. exclusion of the crop, isolation of DNA/RNA, in-hive bees vs. foragers) compared to past studies employing 454 amplicons [32], [33], [35], we still found very similar gut communities when sampling returning pollen foragers. The gut communities of forgers in this study were derived from complete alimentary tracts (guts) of individual bees including the crop, midgut, and hindgut. Tissue specific plate counts for the crop, midgut, and hindgut indicate that the crop bacterial community will not be represented when sequencing the entire alimentary tract using 454-amplicon sequencing (Table 5) [47], [57]. This is because bacteria present at 10^5^–10^6^ (crop) comprise less than 0.01% of the total bacteria in the alimentary tract. For the crop tissues, it was therefore necessary to pool the contents of many foragers per hive to attain sufficient template DNA to overcome potential chance effects that can occur during PCR. Our results agree with [25] that the majority of core gut bacteria are localized in the midgut and hindgut. However, it is clear from this and other studies that core gut bacteria or those typically found in the food stores or pollination environment are also found in the crop with varying frequency.

### Bacteria in the crops of pollen foragers

Our results provide no support for the hypothesis that 13 different beneficial bacteria reside inside the honey crop of bees, are placed on pollen at the flower, and work synergistically to protect beebread from degradation [18]–[24]. Given relatively equal coverage and read number from the crop and gut, we might expect (1) the occurrence of the 13 bacteria at equal or greater frequency in the crop than in the gut, and (2) some level of OTU or sequence read correspondence between the pooled crop and corbicular pollen libraries. A BLAST search of our results reveals that only one of the 13 putative crop bacteria fits both of the above criteria: *Lactobacillus kunkeei*, a species found worldwide in flowers, beehives, many types of bees and their food stores [17], [38], [39], [44], [63]. The 12 remaining “core crop” bacteria occur at much greater frequency in the hindgut than the crop, a pattern consistent with their inclusion in the broad phylogenetic groupings described previously by many independent research groups; *Lactobacillus* sp. Firm 4, *Lactobacillus* sp. Firm 5, and *Bifidobacterium* (Table 4, Fig. 4).

As in [18], [22], our bees were sampled from intentionally untreated, essentially wild colonies. Unfortunately, we cannot provide a direct comparison of our crop abundance measures with published estimates because the methods used to associate plate counts with the abundance of different taxonomic groups in [18] is not evident. The various LAB identified here and in a previous study [17] were morphologically indistinguishable from one another, thus requiring a significant genotyping effort to estimate taxon abundance. As a crude estimate we can use our two crop-specific data sets to estimate the fraction of total crop reads for a given phylotype (Table S2), and express this value as a proportion of the mean plate count for an individual pollen forager crop (7×10^5^ CFU's). This estimate suggests that an average individual forager crop contains 5.6×10^4^ CFU's of *Lactobacillus* Firm 5, 1.8×10^3^ CFU's of *Lactobacillus* Firm 4, and 1.7×10^2^ CFU's of *Bifidobacterium*. As a culture independent comparison, extrapolation from qRT-PCR values based on universal bacterial primers indicates only 10^4^ bacterial 16S rDNA gene copies in the crop [25]. It therefore appears that previous estimates of crop abundance based on plate counts [18], [22] are overestimated by at least an order of magnitude. Based on the sum of available results we conclude that 12 of the 13 putative core crop bacteria are not only inconsistent with the hypotheses detailed earlier [18]–[24], but don't actually occur in the crop or gut with any appreciable frequency or abundance (Table 4, Fig. 4).

In general, the crop contained a microbial community very different from that found in guts (Fig. 3). The crop was dominated by Alpha 2.2 (Acetobacteraceae) and *Lactobacillus kunkeei*, a finding consistent with past results culturing the crops of in-hive bees and honey, as well as culture-dependent and culture-independent assessments of beebread [17], [25]. These two taxa are not considered part of the core gut bacteria, and thrive in sugar-rich, acidic environments such as the crop, beebread and honey [17], [44]. They may be considered core hive bacteria, as they are associated with nurse workers and developing larvae [17], [50]. Alternatively, *L. kunkeei* has been detected worldwide in many different flowers and the pollen provisions of both solitary and social bees suggesting it harbors considerable genetic diversity, and can be readily acquired via the pollination environment [17], [38], [39], [44], [64]. That extremophilic bacteria dominate the crop suggests that it is not an optimal niche for microbial growth, but likely acts as a selective sieve allowing relatively few bacteria to flourish [12], [17]. This conclusion is supported by the consistent differences in bacterial abundance seen among the crops of worker bees performing different tasks (Table 5). In-hive bees contained much lower microbial loads than presumably older bees exposed to the foraging environment. Although crop temperature is fairly constant, and simple sugars likely plentiful, conditions favoring microbial growth in the crop may fluctuate widely according to worker task including pH, osmotic conditions, and the availability protein and other micronutrients.

Almost all crop samples contained the core gut microbiota to some degree (Fig. 3). In most cases, the crop libraries had fewer than 20% of the core gut phylotypes. However, in two of the 14 libraries (colonies 7 and 12), core gut phylotypes contributed 55% and 95% of the crop sequences, respectively. On average, the gut-specific taxa in greatest abundance in the forager crop corresponded to *Lactobacillus* (Firm 5), *Gilliamella apicola* (Gamma1) and *Snodgrassella alvi* (Beta). This contrasts with past culturing results from the crops of newly emerged bees and nurse bees [17]. From that assay, *G. apicola* and *S. alvi* were absent from the crop and grew primarily on pH neutral media, suggesting exclusion from acidic niches and associated media bias. However, the culturing of crop bacteria *L. kunkeei* and Alpha 2.2 were seemingly unaffected by a broad range of pH values. Collectively, this may suggest that the forager crop experiences a reduction in pH, but this remains an open question as foragers are known to differ from younger bees (nurses) for a number of crop and head gland specific components [65]–[67]. Another bacteria considered core to the hindgut, a 97% OTU corresponding to *G. apicola* (OTU 211), was found in the majority of crop libraries with sporadic abundance. It also occurred with much greater abundance in the crop than in the guts, but was not detected with any frequency in corbicular pollen (Table S1). This is an intriguing result as *G. apicola* has been detected at low levels in the crop, and some of the strains can utilize pectin found in the pollen wall [25], [34]. However, neither culturing, cloning, nor qRT-PCR with species-specific primers have detected this taxonomic group in beebread [17], [25].

### Bacteria in the guts of pollen foragers

Most of the recent studies concerning the gut microbiota of honey bees document the bacterial phylotypes in bees that have not yet transitioned to foraging. The forager diet differs from that of a nurse bee. Young (nurse) bees consume large amounts of pollen, producing nutrient rich glandular secretions to feed worker larvae, queens, and older bees [68], [69]. The oldest bees do not ingest pollen, but forage for resources using stored honey and nectar to fuel their flight muscles. Foragers utilize mostly simple sugars, but also receive protein-rich worker jelly at unknown frequency via trophallaxis [61]. Gut microbial communities are strongly affected by host diet [70], [71], and so the results shown in this study add to the existing literature to address how microbial communities change as bees transition to different life stages and diets. Despite diet differences between younger in-hive bees and older foragers, we find that forager guts are, like younger bees, comprised of only a few distinct core phylotypes. After omitting the single deficient gut library for the fall individual 12 we found that every individual forager gut contained *Lactobacillus* (Firm 5), *Snodgrassella alvi* (Beta), *Gilliamella apicola* (Gamma 1), Acetobacteraceae (Alpha 2.1), and *Lactobacillus* (Firm 4). *Bifidobacterium* was detected in 21 of 23 guts. In forager guts collected in the spring and fall, these core bacteria represented 99% and 94% of sequences, respectively. One of three major OTUs corresponding to *Lactobacillus* Firm 5 (050, Table S1) accounted for 12% of Firm 5 reads, is a 100% match to a *Lactobacillus* sp. Firm 5 found in bumble bees [72], and has also been sampled from the apple phyllosphere [73] suggesting some degree of horizontal transmission.

As shown for in-hive bees [33], the relative frequencies of core phylotypes varied considerably among individuals in the same hive and in many cases bacteria identified as core to the gut were not consistently found in foragers. In stark contrast to recent amplicon based work [25], *Frischella perrara* (Gamma 2) was represented as only one read from one forager gut from each sampled time period (Table S1). Further investigation revealed that the primers used in this and other studies [32], [33] were a 100% match to the *F. perrara* 16S rDNA sequence. Thus the finding that foragers contain little to no *F. perrara* relative to younger bees appears authentic. We identified no sequences corresponding to Gamma 4, a Gammaproteobacteria occurring sporadically in previous samples of in-hive bees from this same Arizona location [33].

Many of the individuals with low levels of the core gut microbiota possessed what might be considered environmental bacteria occurring at sporadic abundance consistent with the hypothesis of a disease state. As an example, the gut of fall individual 1 contained the least amount of the core microbiota, but exhibits a high level of non-core Enterobacteriaceae sequences. The putative *Bartonella*-like species Alpha1 (OTU021) and a *Pseudomonas* species (OTU084) also showed a similar pattern in the guts of fall individual 10 and spring individual 14, respectively. That these same bacteria did not occur with any frequency in corbicular pollen suggests their potential as opportunistic gut pathogens, as opposed to recently vectored plant bacteria. In a contrasting pattern, OTU159 classified as *Arsenophonus*, was detected at high frequency from both the crop and corbicular pollen of one pooled colony sample (see [8], [74]).

### Corbicular pollen has a rich microbial community distinct from the gut and crop

Corbicular pollen harbored significantly more taxa than microbial communities of the gut or crop (Table S2). The core gut bacteria were rarely found in corbicular pollen and, on average, comprised less than 3.5% of the corbicular pollen libraries (Fig. 3). In a couple of libraries, the strong presence of core gut bacteria (i.e. 90% in fall individual 4) may have been caused by inadvertently pinching the forager's abdomen with forceps during collection, resulting in contamination of the corbicular pollen pellet with bacteria-rich feces. In general, however, most of the corbicular pollen samples were dominated by bacterial groups often found in wind-blown sediment, soil, flowers, the rhizosphere and phyllosphere such as Gammaproteobacteria, Actinomycetales, and Enterobacteriaceae [42], [75], [76]. Diversity measures indicate that the sampling of corbicular pollen was nearly complete in the fall, whereas spring libraries of corbicular pollen are underestimated. Additionally, it is difficult to determine whether the crop contents simply reflect taxa passing through the crop on their way to the gut and/or whether the crop serves as a source of inoculum for newly collected pollen. Despite these difficulties, informative patterns were evident when comparing OTU frequency and occurrence across microenvironments.

We might infer that OTUs highly abundant in corbicular pollen and either absent or found at much lower frequency in the gut and crop represent bacteria acquired from the pollination environment (Table S2). Microbes abound in the phyllosphere, flowers and nectar of plants, and are highly diverse, often showing strong taxonomic affiliations with their host plant [77]. It is tempting to hypothesize that some of the variation among corbicular pollen samples reflects each individual bees' foraging flight. In a single foraging trip, a worker can collect both nectar and pollen, but foraging typically favors only one of these resources [78]. Individual foragers show strong constancy in the plant species they pollinate and the pollen on a returning forager is frequently derived from a single plant species [79]. In addition, pollen can be inoculated with flower bacteria, and airborne bacteria that stick to the corbicular mass as the bee returns to the hive. Therefore, the bacterial community in corbicular pollen might not only reflect microbes found on the floral resource, but the path traveled by foragers. Abundant corbicular OTUs belonged to environmental bacteria Xanthomonadaceae, Pseudomonadaceae, Enterobacteriacae, Acinetobacter, and many different families of Actinomycetales.

Many of the Actinobacteria uniformly present in individual corbicular pollen samples have also been found at appreciable levels in beebread [17], suggesting they can survive for extended periods in the food stores. Actinobacteria found in beebread were represented by at least 12 families and included a variety of *Streptomyces* [17]. Many Actinobacteria are apparently mutualists, found in the food stores, hive materials, or cuticle of a variety of Hymenoptera including solitary and social bees [49], [80]–[85]. Actinobacteria are renowned for their vast arsenal of metabolic weaponry used to inhibit fungal growth, a common cause of pollen spoilage [37], [86]. Actinobacteria can grow slowly, form spore-like structures, and survive on minimal media [87], such that transmission between hives, environments and life stages may occur on the bodies of individual honey bees. Actinobacteria in corbicular pollen, beebread and brood casings is particularly interesting given the hypothesis that both solitary and social Hymenoptera, developing in close association with resources prone to fungal infection, have strong selective pressure to evolve protective symbioses [88].

Bacteria most abundant in the gut were considered gut-specific, and were consistently found at much lower frequency in the crop and corbicular pollen. These core bacteria may therefore be transmitted to new offspring via trophollaxis, or “leaked” from the crop, as opposed to direct or indirect coprophagy [25]. Their presence in the corbicular pollen could suggest that they function in pollen preservation or nutrition. However, recent cultures, clones, and qRT-PCR with core gut-specific 16S rDNA primers suggest that core gut bacteria do not survive well in beebread. Only two crop inhabiting bacteria, Alpha 2.2 and *Lactobacillus kunkeei*, show a pattern suggestive of corbicular pollen inoculation followed by long term beebread survival [17], [25]. As detailed previously, these two bacteria were much more abundant in the crop than the gut, and also found with considerable frequency in corbicular pollen. Interestingly however, *Lactobacillus kunkeei* was found only in spring corbicular pollen, perhaps due to an environmental or hive-specific effect. In contrast, Alpha 2.2 was consistently abundant in corbicular pollen across both sampling periods. Combined with previous findings, this suggests that Alpha 2.2 is a “core hive” bacterium capable of survival in royal jelly and honey, the most extreme of hive environments [17], [50].

## Conclusion

We catalogued the diversity of bacteria associated with the guts, crops, and corbicular pollen of honey bee foragers. Our results do not support the existing hypothesis of a core crop microbiota comprised of 13 LAB strains [18]–[24]. Rather, when sequences corresponding to these 13 bacterial strains were detected, they were at very low relative frequencies from both the foregut (crop) and entire alimentary tract, with the single exception of *L. kunkeei*, abundant in forager crops. We find additional evidence for a core gut microbiota and add to the existing literature focused on younger in-hive bees [25], [32], [33]. Lastly, we find that corbicular pollen is a microbially diverse environment and likely the source of several plant associated bacteria commonly found in hive food stores.

## Supporting Information

Figure S1 **Rarefaction curves by microenvironment (sample type) and individual library.**
(XLSX)

Table S1 **Barcodes linking the sample types and each individual library to the SRA submission.**
(XLSX)

Table S2 **Number of sequences in each of the 812 OTUs found in guts, crops, and corbicular pollen samples.**
(XLSX)
